# Plasmatic MMP9 released from tumor-infiltrating neutrophils is predictive for bevacizumab efficacy in glioblastoma patients: an AVAglio ancillary study

**DOI:** 10.1186/s40478-021-01305-4

**Published:** 2022-01-03

**Authors:** Carine Jiguet-Jiglaire, Sebastien Boissonneau, Emilie Denicolai, Victoria Hein, Romain Lasseur, Josep Garcia, Sylvie Romain, Romain Appay, Thomas Graillon, Warren Mason, Antoine F. Carpentier, Alba A. Brandes, L.’Houcine Ouafik, Wolfgang Wick, Ania Baaziz, Julien P. Gigan, Rafael J. Argüello, Dominique Figarella-Branger, Olivier Chinot, Emeline Tabouret

**Affiliations:** 1 Aix-Marseille University, CNRS, INP, Inst Neurophysiopathol, Marseille, France; 2 Department of Neursurgery, CHU Timone, Aix Marseille University, APHM, INSERM, MMG, Marseille, France; 3 F. Hoffmann-La Roche Ltd, Basel, Switzerland; 4 APHM, CHU Timone, Service d’anatomopathologie, Marseille, France; 5 Princess Margaret Hospital, Toronto, Canada; 6 APHP, Paris University, Hopital Saint-Louis, Paris, France; 7 Azienda AUSL- IRCCS-Istituto Scienze Neurologiche, Bologna, Italy; 8 APHM, CHU Nord, Service d’Onco-Biologie, Marseille, France; 9 Heidelberg University Medical Center and German Cancer Research Center, Heidelberg, Germany; 10 Aix Marseille University, CNRS, INSERM, CIML, Centre d’Immunologie de Marseille-Luminy, Marseille, France; 11 GlioME Team, APHM, CHU Timone, Service de Neuro-oncologie, Institut de Neurophysiopathologie (INP), CNRS 7051, 27 bd Jean Moulin, 13005 Marseille, France

**Keywords:** Bevacizumab, Glioblastoma, MMP9, Neutrophils, Predictive biomarker

## Abstract

We previously identified matrix metalloproteinase 2 (MMP2) and MMP9 plasma levels as candidate biomarkers of bevacizumab activity in patients with recurrent glioblastoma. The aim of this study was to assess the predictive value of MMP2 and MMP9 in a randomized phase III trial in patients with newly diagnosed glioblastoma and to explore their tumor source. In this post hoc analysis of the AVAglio trial (AVAGlio/NCT00943826), plasma samples from 577 patients (bevacizumab, n = 283; placebo, n = 294) were analyzed for plasma MMP9 and MMP2 levels by enzyme-linked immunosorbent assay. A prospective local cohort of 38 patients with newly diagnosed glioblastoma was developed for analysis of tumor characteristics by magnetic resonance imaging and measurement of plasma and tumor levels of MMP9 and MMP2. In this AVAglio study, MMP9, but not MMP2, was correlated with bevacizumab efficacy. Patients with low MMP9 derived a significant 5.2-month overall survival (OS) benefit with bevacizumab (HR 0.51, 95% CI 0.34–0.76, *p* = 0.0009; median 13.6 vs. 18.8 months). In multivariate analysis, a significant interaction was seen between treatment and MMP9 (*p* = 0.03) for OS. In the local cohort, we showed that preoperative MMP9 plasma levels decreased after tumor resection and were correlated with tumor levels of MMP9 mRNA (*p* = 0.03). However, plasma MMP9 was not correlated with tumor size, invasive pattern, or angiogenesis. Using immunohistochemistry, we showed that MMP9 was expressed by inflammatory cells but not by tumor cells. After cell sorting, we showed that MMP9 was expressed by CD45+ immune cells. Finally, using flow cytometry, we showed that MMP9 was expressed by tumor-infiltrating neutrophils. In conclusion, circulating MMP9 is predictive of bevacizumab efficacy and is released by tumor-infiltrating neutrophils.

**Supplementary Information:**

The online version contains supplementary material available at 10.1186/s40478-021-01305-4.

## Introduction

Glioblastoma is the most aggressive and frequent primary brain tumor in adults. Despite standard first-line treatment based on surgical resection, radiotherapy, and chemotherapy, recurrence remains systematic, with a median delay of 7 to 10 months [1]. At relapse, the use of chemotherapy is associated with response rates ranging from 5 to 15%, whereas the use of immunotherapy or targeted therapy such as epidermal growth factor receptor inhibitors failed to decrease tumor volume or increase patient survival [2]. The use of bevacizumab (Avastin), an antiangiogenic therapy against vascular endothelial growth factor type A (VEGFA), was associated with a response rate of 25% to 35% in patients with recurrent glioblastoma and was evaluated in phase III trials in the initial and recurrent setting [3–5]. In these trials, bevacizumab significantly improved progression-free survival (PFS) of patients at initial diagnosis and at relapse. However, it failed to significantly improve overall survival (OS), restricting its use for patients despite the lack of real effective alternatives. This lack of impact on OS could be related to the heterogeneity of bevacizumab response. The heterogeneity of bevacizumab efficacy, its cost, and its potential side effects, as well as the multiplication of other new therapies under investigation, highlight the need to identify predictive biomarkers of antiangiogenic activity.

In a retrospective study, we previously identified two promising candidates, the plasma levels of matrix metalloproteinase 2 (MMP2) and MMP9, which are potentially able to predict patient response after bevacizumab treatment [6, 7]. In this study, we retrospectively analyzed MMP2 and MMP9 plasma levels in two small, uncontrolled cohorts and observed that MMP2 and, to a lesser extent, MMP9 plasma levels seemed to be associated with patient survival after bevacizumab treatment. The next step regarding these two potential predictive biomarkers was to confirm their predictive value in a prospective, randomized clinical trial evaluating the benefit of bevacizumab versus placebo for glioblastoma patients. MMP9 and MMP2 play an important role in the degradation of extracellular matrix components and are involved in numerous physiological and pathological processes. In cancer, MMP9 and MMP2 appear to play a role in tumor invasion and angiogenesis and to mediate the tumor microenvironment [8, 9]. Degradation of the extracellular matrix by MMP releases proangiogenic compounds, such as vascular endothelial growth factor (VEGF) and integrins [10]. MMP9 has been identified as a biomarker in various cancers, mainly when tumor expression is considered [11]. High expression of MMP2 and MMP9 has been described in glioma, correlated with tumor grade and localized to the cytoplasm of tumor cells, endothelial cells, and their extracellular matrix [12].

In the present study performed in a large prospective international multicentric phase III trial, for the first time to our knowledge, we identified MMP9 plasma level as a biomarker predictive of bevacizumab efficacy in patients with newly diagnosed glioblastoma. Then, exploring the source of MMP in the tumors, we showed that the MMP9 plasma level was significantly higher in glioblastoma patients than in control subjects, decreased after tumor resection, and was correlated with MMP9 tumor expression. However, no correlation was observed between MMP9 and tumor size, invasive pattern, or angiogenesis, in agreement with our results showing that MMP9 was expressed by CD45+ inflammatory cells that we identified as tumor-infiltrating neutrophils but was not expressed by tumor cells.

## Patients and methods

### Patients (Additional file 1: Fig. S1)

#### AVAglio trial cohort (prospective phase III trial)

First, to assess the predictive value of MMP2 and MMP9 plasma levels, we analyzed the plasma samples from the AVAglio trial (NCT 00,943,826). The Avastin in Glioblastoma (AVAglio) study (BO21990) was a randomized, double-blind, placebo-controlled trial sponsored by F. Hoffmann– La Roche (Additional file 1: Fig. S2A) [3]. Briefly, patients 18 years of age or older with newly diagnosed, histologically confirmed, supratentorial glioblastoma were randomly assigned, in a 1:1 ratio, to bevacizumab or placebo. All patients were required to give written informed consent before enrollment. The protocol was approved by the applicable independent ethics committees and institutional review boards. The study adhered to the principles of the Declaration of Helsinki and the Guidelines for Good Clinical Practice. After undergoing surgical resection or biopsy, patients received concurrent radiotherapy and oral temozolomide (Additional file 1: Fig. S2A) in combination with intravenous bevacizumab (BEV) or placebo every 2 weeks. In the maintenance phase, patients received temozolomide plus intravenous bevacizumab or placebo every 2 weeks, for six 4-week cycles. In the monotherapy phase, intravenous bevacizumab (15 mg per kilogram) or placebo was continued every 3 weeks until the disease progressed, or unacceptable toxic effects developed. The determination of progression was based on imaging assessment (MRI), clinical assessment, and glucocorticoid use. Plasma samples were collected at baseline, within 24 to 48 days after surgery and prior the administration of any study treatment. Peripheral blood was drawn into EDTA tube. Blood samples were centrifuged within 2 h at 3000 × *g* for 10 min at room temperature and plasma was stored at −80 °C.

#### Prospective peri-operative patient cohorts

To understand the relationship between plasmatic MMP9 and glioblastoma, we established a local prospective cohort composed by 38 adult (≥ 18 years) *IDHwt* glioblastoma patients included at initial diagnosis between June 2016 and October 2017 at Timone Hospital (Marseille, France). For these patients, the plasma samples were collected before and 48 h after surgical resection, as well as paraffin and frozen tumor samples. A concomitant MRI was performed at the time of diagnosis including T1, T1 with gadolinium, T2, FLAIR and perfusion sequences. Tumor volumes were analyzed using the Horos software®. Perfusion sequences were analyzed using the Olea Medical software®. This cohort was completed by 7 patients hospitalized for the resection of brain aneurysm and 12 healthy controls.

The following data were recorded: age, gender, type of surgery, Karnofsky Performance Status (KPS), oncological treatment, clinical symptom, steroid dose, and MRI characteristics.

All samples were stored in the APHM Biological Resource Center (BRC) (authorization number AC2018-31053; CRB BB-0033-00097). Tumor and plasma samples were obtained after written consent according to a protocol approved by the local institutional review board and ethics committee. The present studies were conducted in accordance with the declaration of Helsinki.

### Methods

#### Enzyme-linked immunosorbent assay (ELISA)

Plasma samples stored at −80 °C were diluted and assayed with sandwich ELISA for MMP2, MMP9, VEGFA, VEGFR2, CXCL12 (Quantikine ELISA Kit from R&D Systems) and CXCR4 (CUSABIO) as recommended by manufacturers. Protein quantities were calculated with a calibrated specific standard.

#### Immunohistochemistry (IHC)

Five micrometers formalin fixed paraffin embedded (FFPE) slides of tumor samples were labeled with anti-MMP2 or anti-MMP9 antibodies (MMP2, ab37150, 1/25—MMP9, ab38898 Abcam, 1/1000). Staining was performed on a Benchmark XT (Ventana Medical systems, Illkirch, France) according to manufacturer's instructions. A semi-quantitative analysis was done by two pathologists (RA, DFB) who read all samples together, to define the expression location and level (from 0: absent to 3: high) without knowledge of clinical data.

#### Immunofluorescence (IF)

Five µm frozen section of tumor samples were permeabilized with ethanol-acetone (19v–1v) before saturation in PBS- BSA5%. Sequential staining with anti-CD31/anti-MMP9 antibodies or anti-CD45/anti-MMP9 antibodies were done (CD31, JC70A, Dako, 1/50—CD45, HI30, Invitrogen, 1/200—MMP9, ab38898 Abcam, 1/1000). Secondary antibodies were purchased from Molecular Probes™, goat anti mouse-AlexaFluor 488 (A11001) for antibodies targeting CD31 and CD45 and goat anti rabbit-AlexaFluor 568 (A11011) for anti-MMP9 antibody. Nuclei were stained with Hoechst 33,342 (B2261, Sigma, 1/1000). Fluorescent microcopy pictures were done with Zeiss® Observer. Z1 and ZenPRO software and analysis were done with ImageJ software.

#### Magnetic sorting

Fresh glioblastoma samples were obtained from APHM—Biological Resource Center (AC-2018-31053; CRB BB-0033-00097)—after surgical resection. Tissues were first dissociated with Brain tumor dissociation kit as recommended by manufacturer. Myelin was excluded from cell suspension with Myelin Isolation Beads. Part of the whole tumor was set aside as a future control. Then, magnetic sorting of CD31^+^ or CD45^+^ cells was done with specific microbeads (CD31 MicroBead Kit and CD45 MicroBeads from Miltenyi Biotec). Cell fraction purity was assayed by flow cytometry. Samples were validated for further analysis if purity reached 65%.

#### Flow cytometry analysis

All analyses were done on fresh newly-diagnosed glioblastoma samples. For the initial analyses (Fig. 4), cells were stained for extracellular proteins (CD31-APC, clone REA730, 1/50; CD45-APC, clone HI30, 1/5, BD biosciences). After fixation, cells were permeabilized with 0.1% saponin before MMP staining (MMP2-PE, clone 1A10, 1/10; MMP9-FITC, clone 56,129, 1/10, R&Dsystems). Cell staining were analysed on a BD FACSCalibur™ cytometer and CellQuest software.

For the second analyses (Fig. 5), flow cytometry was conducted using BD Symphony and BD LSR Fortessa X-20 machine (BD Biosciences™) and data were analyzed with FlowJo (Tree Star™). Tumor cell suspentions were stained with LIVE/DEAD™ Fixable Aqua Dead Cell Stain (Invitrogen™, Catalog No. L34957) to stain dead cells, Fc blocked with anti-CD16/32 cells and fixed/RBC lysed with RBC Lysis/Fixation Solution (10X) (Biolegend Cat. No. 422401) and the following anti-Human antigens antibodies were used for staining glioblastoma derive single cell suspensions:

BUV395 Mouse Anti-Human CD11c (Clone B-ly6, BD Bioscience, Cat. No. 563787), BUV496 Mouse Anti-Human CD3 (Clone UCHT1, BD Bioscience, Cat. No. 564809), BUV737 Mouse Anti-Human CD64 (Clone 10.1, BD Bioscience, Cat. No. 612776), BUV805 Mouse Anti-Human CD14 (Clone M5E2, BD Bioscience, Cat. No. 612902), eFluor 450 Mouse Anti-Human CD123 (Clone 6H6, eBioscience, Cat. No. 48-123-42), BV605 Anti-Human CD8a (Clone RPA-T8, BioLegend, Cat. No. 301040), BV650 Mouse Anti-Human CD163 (Clone GHI/61, BD Bioscience, Cat. No. 563888), BV786 Mouse Anti-Human gdTCR (Clone 11F2, BD Bioscience, Cat. No. 744743), PE Mouse Anti-Human CD16 (Clone RUO, BD Bioscience, Cat. No. 555407), PE-Dazzle Mouse Anti-Human CD197 (CCR7) (Clone G043H7, Biolegend, Cat. No. 353236), APC Anti-Human HLA-DR (Clone REA805, Miltenyi Biotec, Cat. No. 130-111-943), APC-R700 Rat Anti-Human CXCR5 (Clone RF8B2, BD Bioscience, Cat. No. 555407), APC-eFluor 780 Mouse Anti-Human CD45(Clone HI30, eBioscience, Cat. No. 47-0459-42). After washing cells were fixed and permeabilized with the Foxp3/Transcription Factor Staining Buffer set (eBioscience Cat#00-5323-00), washed and stained with FITC Mouse Anti-Human MMP9 (Clone 56,129, R&D Systems, Cat. No. IC9111F), PerCP-Cy5.5 Mouse Anti-Human perforin (Clone G9, BD Bioscience, Cat. No. 563762).

#### Real-time quantitative polymerase chain reaction (RT-qPCR)

*MMP2 and MMP9* gene and *18S*, *B-actin* and *GAPDH* reference gene expressions were assayed by RT-qPCR (Additional file 2: Table S1). Total RNA was reverse transcribed and target genes were quantified by amplification using the Light Cycler Real- Time PCR (Roche Applied Science, Meylan, France). RT-qPCR was performed by using a medium containing 1X LightCycler 480 SYBR Green I master mix, 0.25 μM of each primer and 20 ng of cDNA. Each PCR reaction was preceded by one activation cycle of 95 °C for 5 min and ended by establishing a melting curve 5 degrees above the oligonucleotide melting temperature.

#### Statistical analyses

Categorical variables were presented as frequencies and percentages, continuous variables as median, quartile and range. Overall survival (OS) was defined to be time from randomization to death from any cause, censored at the date of last contact. Progression free survival (PFS) was defined to be the time from randomization to documented progression or death, censored at the date of the last documented disease evaluation. The Kaplan–Meier method was used to estimate survivals distributions. Log-rank tests were used for univariate comparisons. Cox proportional hazards models were used for multivariate analyses and to estimate hazard ratios in survival regression model. Interaction test was done between biomarker subgroups and treatment arms. Subjects were divided into four groups based on their baseline biomarkers levels using the quartile value as the cutoff. Mann–Whitney U-test or Kruskal–Wallis test were used to compare quantitative values; qualitative values were analyzed using Fisher exact test and Chi 2 test. Correlations were analyzed using the Spearman test. All reported *p*-values are two-sided, and *p* < 0.05 was considered statistically significant. This study followed the REMARK criteria. All statistical analyses were performed using SPSS software v22 ®.

## Results

### Plasma MMP9 predicts bevacizumab efficacy in patients with newly diagnosed glioblastoma: AVAglio ancillary study

**Fig. 1:**
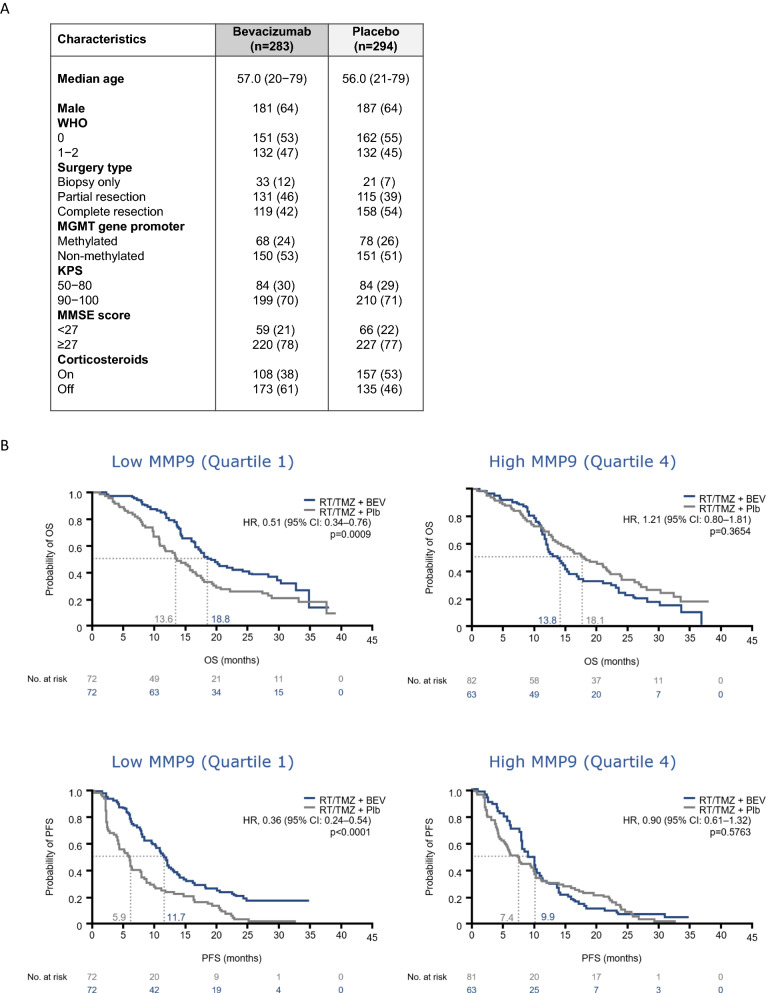
MMP9 plasma level is predictive of bevacizumab efficacy (AVAglio trial). **A** Patient characteristics in the two arms of AVAglio ancillary sub-population. **B** Overall Survival (OS, top) and Progression Free Survival (PFS, bottom) according to low plasma level of MMP9 (left) or high plasma level of MMP9 (right) at baseline. RT: radiotherapy; TMZ: temozolomide; BEV: bevacizumab; Plb: placebo

To assess the predictive value of plasma MMP2 and MMP9 levels, we analyzed 577 of 921 patients included in the AVAglio trial for whom plasma samples were available at inclusion: 283 patients treated by radiochemotherapy and bevacizumab and 294 patients treated by radiochemotherapy and placebo. Patient characteristics were similar between the two treatment groups (Fig. 1A and Additional file 2: Tables S2 and S3). Overall survival observed in our subpopulations were comparable to those reported in the overall population (Additional file 1: Fig. S2B and S2C). The median MMP9 levels at baseline were similar in the placebo and bevacizumab arms: 82.4 ng/mL (44.8–160.3) and 83.6 ng/mL (43.8–133.8), respectively. Patients with low MMP9 plasma levels (< quartile 1) presented with a significant OS benefit from bevacizumab (HR 0.51, 95% CI 0.34–0.76, *p* = 0.0009), which translated into a median increase in OS of 5.2 months (Fig. 1B); a consistent benefit in PFS was also seen (HR 0.36, 95% CI 0.24–0.54, *p* < 0.0001). Interestingly, in patients with high MMP9 (> Q3), OS tended to be longer in the placebo arm (HR 1.21, 95% CI 0.80–1.81). In multivariate analysis including enzyme-inducing antiepileptic drugs, MMSE, *MGMT* promoter methylation, World Health Organization performance status, race, and age, a significant interaction was seen between treatment group and MMP9 plasma level for OS (*p* = 0.0089) and PFS (*p* = 0.0045). In contrast to our previous study, MMP2 did not have predictive value (Additional file 1: Fig. S3). Importantly, MMP2 and MMP9 plasma levels were not prognostic of PFS or OS. Taken together, these results suggest that baseline plasma MMP9 level was predictive of the benefits of bevacizumab for PFS and OS in patients with newly diagnosed glioblastoma.

### Circulating MMP9 is specifically released by glioblastoma

**Fig. 2:**
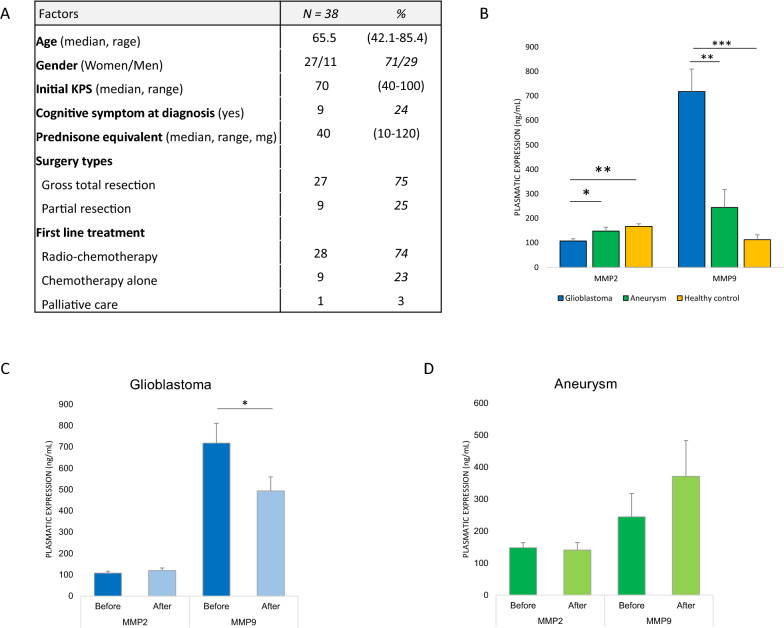
Plasmatic expression of MMP9 is specific for glioblastoma. **A** Patient characteristics of peri-operative cohort. **B** Plasmatic expression of MMP2 and MMP9 before surgical resection of newly-diagnosed glioblastoma or cerebral aneurysm and in healthy controls. **C** MMP2 and MMP9 plasma level before and after glioblastoma resection. **D** MMP2 and MMP9 plasma level before and after cerebral aneurysm resection. MMP9 is highly expressed into glioblastoma and significantly decreases after tumor resection. *p < 0.05; **p < 0.01; ***p < 0.001

To explore the relationship between MMP9 plasma level and intratumoral glioblastoma biology, we analyzed the plasma expression of this marker before and after surgical resection of glioblastoma and its concomitant tumor expression in a prospective cohort of 38 patients (Fig. 2A–D).

First, we showed that the baseline MMP9 plasma level was higher in glioblastoma patients than in patients operated for cerebral aneurysm or healthy controls (*p* = 0.006) (Fig. 2B). Then we observed that the MMP9 plasma level significantly decreased after glioblastoma resection (*p* = 0.03) (Fig. 2C). In contrast, the MMP9 plasma level was not changed after aneurysm resection (Fig. 2D). Finally, we observed that the presurgical plasma MMP9 level was significantly correlated with MMP9 mRNA tumor expression (*p* = 0.033). Of note, the MMP2 plasma level was lower in glioblastoma patients than in aneurysm or control patients, was not correlated with MMP2 tumor expression, and did not vary during the perioperative time.

To explore MMP2 and MMP9 activity, we performed zymogram analyses. MMP9 activity tended to decrease after glioblastoma resection (Additional file: Fig. S4), while no modification was observed after aneurysm resection.

Finally, to explore the involvement of MMP9 in angiogenic and invasive processes, we concomitantly analyzed the baseline plasma expression of five related factors implicated in the tumor microenvironment: MMP2, VEGFA, VEGFR2, CXCR4, and CXCL12. The plasma level of MMP9 was not correlated with these other markers (data not shown), suggesting that MMP9 was independent of these angiogenic and invasive processes.

Taken together, these results suggest that plasma MMP9 is specifically released by glioblastoma, is not only linked to brain alteration and is not correlated to angiogenic or invasive markers.

### MMP9 is not correlated with glioblastoma tumor volume, invasion, or angiogenesis assessed by neuro-imaging

**Fig. 3:**
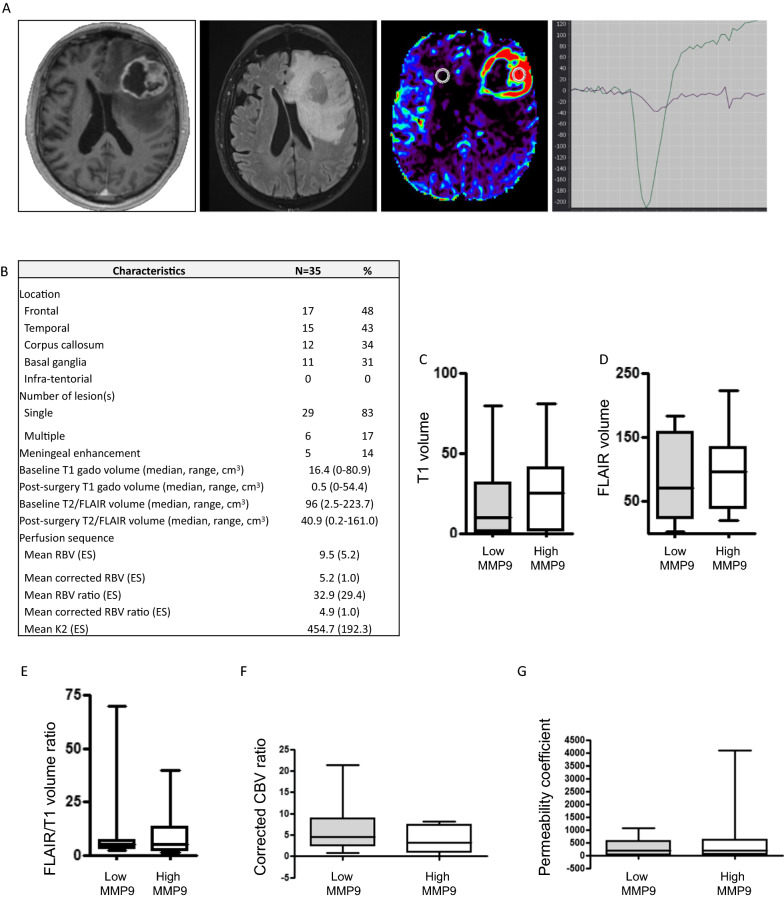
Correlation between MMP9 plasma level and neuro-radiological characteristic. **A **Illustrative magnetic resonance imaging (MRI) showing (from left to right): T1 sequence with contrast enhancement, FLAIR sequence, perfusion sequence and perfusion curve. Round mark: Region Of Interest (ROI) in enhanced tumor (right) and controlateral brain (left, control). **B** Neuro-radiological characteristics of peri-operative cohort. **C** T1 volume according to MMP9 plasma level. **D** FLAIR volume according to MMP9 plasma level. **E** Infiltrative pattern of glioblastoma (using the FLAIR/T1 volume ratio) according to MMP9 plasma level. **F** Corrected CBV ratio according to MMP9 plasma level. **G** Permeability coefficient according to MMP9 plasma level. No significant difference was shown

We then analyzed the correlations between baseline circulating MMP9 levels and the neuroimaging pattern of patients before adjuvant treatment (Fig. 3). All patients presented with supratentorial glioblastoma. Most of them presented with a single lesion at diagnosis (Fig. 3B). The median baseline T1 enhancement and T2/FLAIR volumes were 16.4 cm^3^ and 96 cm^3^_,_ respectively (Fig. 3B). Because the MMP9 plasma level was correlated with MMP9 tumor expression, we analyzed the correlation between plasma MMP9 level and tumor volume. No correlation was found between MMP9 plasma level and enhancement volume (T1 with gadolinium) or tumor infiltration volume (T2/FLAIR sequences) (Fig. 3C, D). Then, because MMP9 was reported to be involved in invasive processes through extracellular matrix remodeling, we analyzed the infiltrative pattern of glioblastoma using the ratio enhancement volume/FLAIR volume. This ratio was not correlated with MMP9 plasma level (Fig. 3E). Finally, no correlation was found between circulating levels of MMP9 and perfusion characteristics (Fig. 3F, G). Regarding the MMP9 activity evaluated by zymogram, no correlation was observed between MMP9 activity and FLAIR volume (*p* = 0.284), contrast enhancement volume (*p* = 0.325) or infiltrative pattern, as previously defined (*p* = 0.774). Of note, no correlation was observed between MMP2 plasma level and neuroimaging characteristics (data not shown). These results suggest that tumor growth, invasion, and vascularization seem to be independent of MMP9 level, suggesting that MMP9 could be related to another glioblastoma microenvironment process.

### MMP9 is expressed by immune cells of the glioblastoma microenvironment

**Fig. 4:**
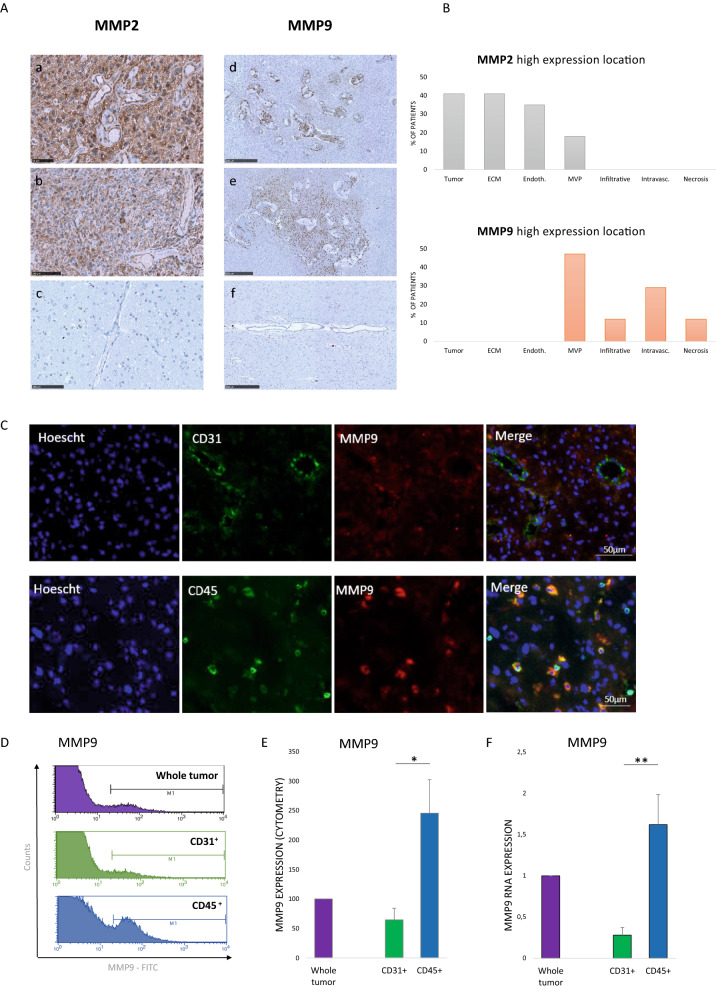
Sub-histological location of MMP2 (left) and MMP9 (right) expression in glioblastoma tissue. **A** Immunostaining of MMP2 expression (left) on glioblastoma (a, b) and cortectomy (c) tissues. Immunostaining of MMP9 expression (right) on glioblastoma (d, e) and cortectomy (f) tissues. Scale bars: a: 50 µm; b-c: 100 µm; d-f: 250 µm. **B** Semi-quantitative analysis of MMP2 and MMP9 immunostaining (high expression: score 2 or 3). Tumor: tumor cells; ECM: extracellular matrix; Endoth.: endothelial cells; MVP: microvascular proliferation; Infiltrative: infiltrative cells; Intravasc.: intravascular cells; Necrosis: necrosis cells. MMP9 is expressed by CD45+ cells in glioblastoma patient samples. **C** Immunofluorescent staining of CD31, CD45 and MMP9 on frozen glioblastoma samples. MMP9 is expressed by CD45 positive cells. Nuclei were stained by Hoechst. Scale bar: 50 µm.** D** Count of MMP9 positive cells in different tumor fractions. Tumors were processed to isolate individual cells; immunomagnetic sorting allowed to separate cells for CD31^+^ and CD45^+^ expression. Each fraction was analyzed for MMP9 expression by cytometry. MMP9 is significantly overexpressed by CD45^+^ fraction. **E** Relative expression of MMP9 by CD31 + and CD45+ fraction cells by cytometry (N = 3). F- MMP9 mRNA expression in CD31 + and CD45+ fraction cells. MMP9 mRNA expression is significantly higher in CD45^+^ fraction (N = 5). *p < 0.05; **p < 0.01

To determine the origin of MMP9 in glioblastoma, we first analyzed its expression by immunohistochemistry (Fig. 4A, B, Additional file 1: Fig. S5). At initial diagnosis, MMP9 staining was mainly located in the microvascular proliferation, inflammatory cells, and circulating intravascular cells (Fig. 4A, B). No staining was observed in glioblastoma cells or in the extracellular matrix. In contrast, MMP2 staining was mainly located in tumor cells and the extracellular matrix (Fig. 4A, B).

Then, to characterize which cells express MMP9, we analyzed the coexpression of MMP9 with the vascular marker CD31 and the immune marker CD45 by immunofluorescence (Fig. 4C). Immunofluorescence staining of MMP9 confirmed its predominant expression in the perivascular area, but MMP9 was not coexpressed by CD31 + vascular cells. In contrast, most cells (> 80%) that were positive for MMP9 coexpressed CD45 (Fig. 4C). To confirm that MMP9 was expressed by immune cells, we sorted the CD31 + cells from fresh glioblastoma samples and the CD45+ cells from the remaining tissue to analyze their MMP9 protein expression by cytometry (N = 3; Fig. 4D, E) and their MMP9 and MMP2 mRNA expression by reverse transcriptase-quantitative polymerase chain reaction (RT-qPCR) (N = 5; Fig. 4F and Additional file 1: Fig. S6). As expected, MMP9 was mainly expressed at the protein and mRNA levels by the CD45+ populations. Taken together, these results confirm that MMP9 is indeed expressed by immune cells infiltrating the glioblastoma microenvironment.

### MMP9 is mainly expressed by glioblastoma-infiltrating neutrophils (Fig. 5 and Additional file 1: Fig. S7)

**Fig. 5:**
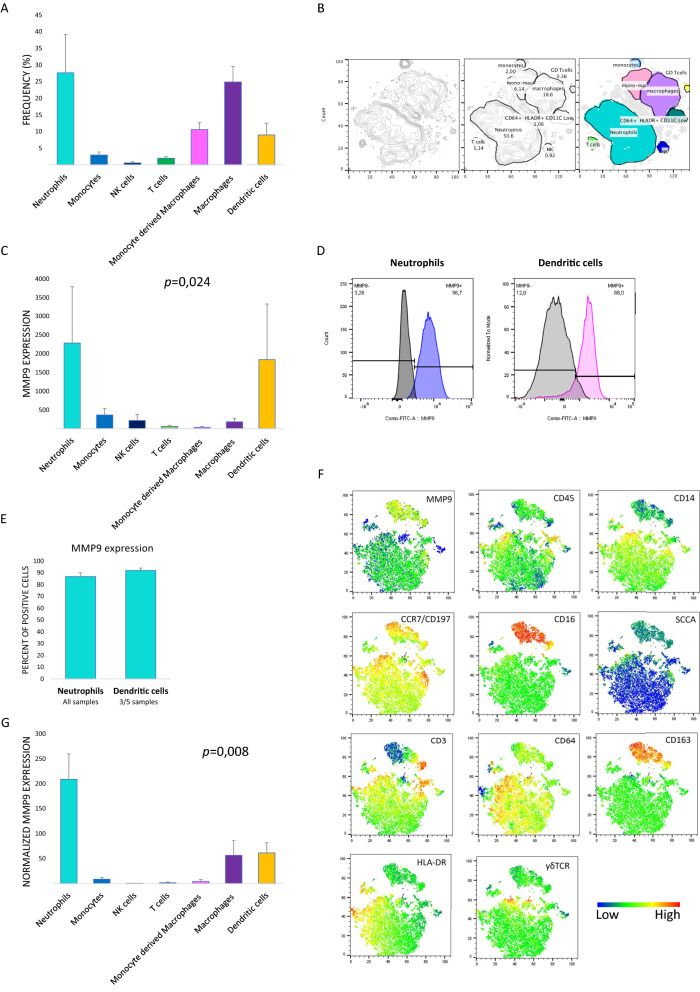
MMP9 is mainly expressed by tumor-infiltrating CD16 + neutrophils. **A** Frequency of immune subsets into the CD45+ cells.** B** T-SNE representation of immune subset in an illustrative case.** C** MMP9 expression: delta (d) Mean Fluorescence Intensity (MFI) [MFI mmp9—MFI fluorescence minus one (fmo)] in each immune subset.** D** MMP9 mean fluorescence intensity (MFI) shift between stained and unstained neutrophils (left) and inflammatory dendritic cells (right).** E** Percent of positive neutrophils (present in all cases) and inflammatory dendritic cells (present in only 3/5 cases) for MMP9.** F** T-SNE representation of the markers including MMP9 in an illustrative case.** G** Normalized expression of MMP9 based on CD45+ cell frequency and dMFI [%CD45*dFMI]

To determine which immune population was responsible for MMP9 expression in the glioblastoma microenvironment, we analyzed five fresh *IDHwt* glioblastoma samples by flow cytometry using a large panel of antibodies. Neutrophils and macrophages represented the main CD45+ subsets (Fig. 5A, B). Inflammatory dendritic cells were only present in 3 samples. The maximal mean fluorescence intensity shift between stained and unstained samples for MMP9 was observed for neutrophils and dendritic cells (*p* = 0.024, Fig. 5C, D). Neutrophils and inflammatory dendritic cells (when present) were positive for MMP9 in 87% and 92% respectively (Fig. 5E, F). After normalization by cell frequency and mean fluorescence intensity shift, the neutrophils appeared to be the main origin of MMP9 expression in *IDHwt* glioblastoma (*p* = 0.008, Fig. 5G). Taken together, these results suggest that the largest amount of MMP9, which seems to be involved in resistance to bevacizumab, is expressed by the tumor-infiltrating neutrophils.

## Discussion

In the present post hoc analysis of a phase III trial (AVAglio), we showed that baseline MMP9 plasma level is predictive of bevacizumab efficacy in patients with newly diagnosed glioblastoma. Then, we showed that MMP9 plasma level is correlated with glioma tissue RNA level and is released by tumor-infiltrating neutrophils from the glioblastoma microenvironment.

These results represent an important contribution to neuro-oncology. For the first time, to our knowledge, we were able to validate an accessible predictive biomarker for bevacizumab activity in glioblastoma patients in a prospective phase III trial. Identifying a predictive biomarker of antiangiogenic therapy is an unmet oncological need. Antiangiogenic therapies improve patient outcomes, but the responses remain heterogeneous, resulting in heterogeneous use and approval restrictions. The clinical benefit for glioblastoma patients associated with bevacizumab motivated approval of the drug in numerous countries, including the United States, but not in Europe due to the lack of survival benefit in phase III trials [3, 4]. However, considering that glioblastoma remains one of the most aggressive and lethal tumors, with limited therapeutic options, identification of patients who could benefit from bevacizumab would considerably improve their therapeutic opportunities. Several predictive candidates were previously tested in oncology: tissue biomarkers (VEGFA, VEGFR2, CAIX, HIF2α, and hERG1) [13], molecular biomarkers (HIF1α, VEGFA, VEGF single nucleotide polymorphisms, transcriptomic signature, microRNA signature, angiotensinogen promoter methylation) [14, 15], circulating biomarkers (plasma levels of VEGFA, CAIX, Tie2, and bevacizumab trough concentration) [16–19], or clinical factors (hypertension) [20]. In most studies, these markers were only prognostic. Interestingly, some inflammatory markers were also investigated, such as CD8 + T-cell count, neutrophil count, neutrophil-to-lymphocyte ratio, and C-reactive protein or interleukin 6 plasma levels [21–24]. We recently identified MMP2 and MMP9 plasma levels as two promising biomarkers of bevacizumab activity. MMP2 and, to a lesser extent, MMP9 were predictive of response, PFS, and OS in two independent small cohorts of patients with recurrent high-grade gliomas treated with bevacizumab, but not in a third cohort of patients treated by chemotherapy only [6]. Here, in contrast to our previous study, the predictive value of MMP9 is clearly identified, whereas no predictive value could be attributed to MMP2. Of note, in the present study, a trend was observed for a potential detrimental effect of bevacizumab use in patients with a high MMP9 level, which reinforces our results. The status of the disease (recurrent versus newly diagnosed) and the patient’s dataset issue from a large prospective randomized study may contribute to the differences between these two studies. We also showed that these biomarkers were correlated with disease control under treatment with plasma level modifications at the time of recurrence [7]. Taken together, these results would allow the use of these biomarkers both at baseline, to select responders, and during treatment, to identify bevacizumab escape early. Advantages of these markers are that they are accessible (a simple blood test), inexpensive, fast, easy to use, and reproducible. Validation of the predictive value of MMP9 in a prospective trial is a major step toward its potential routine clinical use. These results raise new questions about the appropriate cutoff for clinical use and the potential use of MMP9 with other antiangiogenic agents and in other tumor types.

To decipher the link between circulating MMP9 and the response of glioblastoma to bevacizumab, we first showed that circulating MMP9 was dependent on glioblastoma, because circulating MMP9 decreased after glioblastoma resection, was correlated with MMP9 expression in the tumor, and was specific to glioblastoma as compared with brain injury. Then, we showed that MMP9 was mainly released by the tumor-infiltrating neutrophils of the glioblastoma microenvironment. MMP9 expression and activity were previously reported to be preferentially displayed by high-grade gliomas [25] and were suspected to be prognostic [26]. Moreover, a correlation between plasma expression of MMP9 and anaplastic glioma activity was previously reported, reinforcing the link between this circulating marker and the glioblastoma process [27]. Interestingly, our study showed that plasma MMP9 was not prognostic of tumor progression but was specifically predictive of bevacizumab response. The potential implication of MMP9 in antiangiogenic prediction was previously suggested in two case reports that reported increasing urinary MMP9 activity at the time of antiangiogenic escape [28]. The pattern of expression of MMP9 in glioblastoma remains controversial. In other tumor types, MMP9 was previously reported to be expressed by immune cells, such as pathogenic M2 macrophages [29, 30], tumor-infiltrating neutrophils [31, 32] or myeloid-derived suppressor cells [33]. In gliomas, previous studies suggested that MMP9 could be released by microglial cells [34], macrophages [35], or neutrophils [26]. In our study, we used for the first time a large cytometry panel to refine the expression profile of MMP9 in five fresh tumor samples at diagnosis and observed that MMP9 was mainly released by tumor-infiltrating neutrophils. Regarding the involvement of neutrophil-released MMP9 and resistance to antiangiogenic therapy, Nozawa et al*. *[36] showed in a pancreas model that tumor-infiltrating neutrophils mediated the angiogenic switch by releasing MMP9, which was able to degrade the extracellular matrix, thus making VEGFA available by proteolytic cleavage. Deryugina et al. [37] reported similar results with complementary preclinical models of systemic cancers. The proangiogenic role of neutrophils through MMP9 expression is further supported by the description of a specific proangiogenic neutrophil subpopulation, which was distinct from inflammatory neutrophils recruited to infectious sites, releasing a larger amount of MMP9 in a pancreatic mouse model [38]. Finally, independently of the immune system, the involvement of MMP9 in VEGFA release was also reported in a preclinical model of glioblastoma, highlighting its potential central role in the initiation and promotion of angiogenesis [39, 40]. On the basis of these preclinical results, we can hypothesize that MMP9 released by tumor-infiltrating neutrophils may increase VEGFA availability in the glioblastoma microenvironment, exceeding the ability of bevacizumab to trap it. In the context of the failure of classical immunotherapy in glioblastoma patients, this specific neutrophil population could represent a promising target for specific immunomodulation in neuro-oncology.

Our study has some limitations. Our study lack of an independent validation set to confirm our findings: we would need to validate them in an independent prospective randomized trial. Moreover, the second peri-operative cohort is only composed by 38 patients. The conclusions issued from this second cohort are limited by its size and should be carefully interpreted as hypotheses. The confirmation of these results in a larger cohort is mandatory as well as preclinical exploration of the mechanistical implication of tumor-infiltrating neutrophils in MMP9 expression and bevacizumab sensibility.

In conclusion, the baseline plasma level of MMP9 may be predictive of the efficacy of bevacizumab for patients with newly diagnosed glioblastoma. Circulating MMP9 is released by glioblastoma-infiltrating neutrophils, suggesting that antiangiogenic resistance could be related to a specific immunological glioblastoma profile, thus opening new perspectives in the development of therapy for glioblastoma.

## Supplementary Information

**Additional file 1**. Supplemental figures.

**Additional file 2**. Supplementary tables.

## Data Availability

The data that support the findings of this study are available from Roche® but restrictions apply to the availability of these data, which were used under license for the current study, and so are not publicly available. Data are however available from the authors upon reasonable request and with permission of Roche®.

## References

[1] Stupp R, Mason WP, van den Bent MJ, Weller M, Fisher B, Taphoorn MJB (2005). Radiotherapy plus concomitant and adjuvant temozolomide for glioblastoma. N Engl J Med.

[2] Weller M, van den Bent M, Preusser M, Le Rhun E, Tonn JC, Minniti G (2021). EANO guidelines on the diagnosis and treatment of diffuse gliomas of adulthood. Nat Rev Clin Oncol.

[3] Chinot OL, Wick W, Mason W, Henriksson R, Saran F, Nishikawa R (2014). Bevacizumab plus radiotherapy-temozolomide for newly diagnosed glioblastoma. N Engl J Med.

[4] Wick W, Gorlia T, Bendszus M, Taphoorn M, Sahm F, Harting I (2017). Lomustine and bevacizumab in progressive glioblastoma. N Engl J Med.

[5] Gilbert MR, Dignam JJ, Armstrong TS, Wefel JS, Blumenthal DT, Vogelbaum MA (2014). A randomized trial of bevacizumab for newly diagnosed glioblastoma. N Engl J Med.

[6] Tabouret E, Boudouresque F, Barrie M, Matta M, Boucard C, Loundou A (2014). Association of matrix metalloproteinase 2 plasma level with response and survival in patients treated with bevacizumab for recurrent high-grade glioma. Neuro-Oncol.

[7] Tabouret E, Boudouresque F, Farina P, Barrié M, Bequet C, Sanson M (2015). MMP2 and MMP9 as candidate biomarkers to monitor bevacizumab therapy in high-grade glioma. Neuro-Oncol.

[8] Kessenbrock K, Plaks V, Werb Z (2010). Matrix metalloproteinases: regulators of the tumor microenvironment. Cell.

[9] Bruni-Cardoso A, Johnson LC, Vessella RL, Peterson TE, Lynch CC (2010). Osteoclast-derived matrix metalloproteinase-9 directly affects angiogenesis in the prostate tumor-bone microenvironment. Mol Cancer Res MCR.

[10] Rundhaug JE (2005). Matrix metalloproteinases and angiogenesis. J Cell Mol Med.

[11] Huang H (2018). Matrix metalloproteinase-9 (MMP-9) as a cancer biomarker and MMP-9 biosensors: Recent advances. Sensors.

[12] Wang M, Wang T, Liu S, Yoshida D, Teramoto A (2003). The expression of matrix metalloproteinase-2 and -9 in human gliomas of different pathological grades. Brain Tumor Pathol.

[13] Iorio J, Lastraioli E, Tofani L, Petroni G, Antonuzzo L, Messerini L (2020). hERG1 and HIF-2α behave as biomarkers of positive response to bevacizumab in metastatic colorectal cancer patients. Transl Oncol.

[14] Faloppi L, Puzzoni M, Casadei Gardini A, Silvestris N, Masi G, Marisi G (2020). Angiogenesis genotyping and clinical outcomes in patients with advanced hepatocellular carcinoma Receiving Sorafenib: The ALICE-2 study. Target Oncol.

[15] Yan C, Wang J, Yang Y, Ma W, Chen X (2019). Molecular biomarker-guided anti-angiogenic targeted therapy for malignant glioma. J Cell Mol Med.

[16] Soyama H, Miyamoto M, Matsuura H, Iwahashi H, Kakimoto S, Ishibashi H (2020). Rapid decrease in serum VEGF-A levels may be a worse prognostic biomarker for patients with platinum-resistant recurrent ovarian cancer treated with bevacizumab and gemcitabine. Cancer Chemother Pharmacol.

[17] Janning M, Müller V, Vettorazzi E, Cubas-Cordova M, Gensch V, Ben-Batalla I (2019). Evaluation of soluble carbonic anhydrase IX as predictive marker for efficacy of bevacizumab: a biomarker analysis from the geparquinto phase III neoadjuvant breast cancer trial. Int J Cancer.

[18] Jayson GC, Zhou C, Backen A, Horsley L, Marti-Marti K, Shaw D (2018). Plasma Tie2 is a tumor vascular response biomarker for VEGF inhibitors in metastatic colorectal cancer. Nat Commun.

[19] Papachristos A, Kemos P, Kalofonos H, Sivolapenko G (2020). Correlation between bevacizumab exposure and survival in patients with metastatic colorectal cancer. Oncologist.

[20] Tabouret E, Chinot O, Sanson M, Loundou A, Hoang-Xuan K, Delattre J-Y (2014). Predictive biomarkers investigated in glioblastoma. Expert Rev Mol Diagn.

[21] Kalathil SG, Hutson A, Barbi J, Iyer R, Thanavala Y (2019). Augmentation of IFN-γ+ CD8+ T cell responses correlates with survival of HCC patients on sorafenib therapy. JCI Insight.

[22] Quillien V, Carpentier AF, Gey A, Avril T, Tartour E, Sejalon F (2019). Absolute numbers of regulatory T cells and neutrophils in corticosteroid-free patients are predictive for response to bevacizumab in recurrent glioblastoma patients. Cancer Immunol Immunother CII.

[23] Li B, Wang S, Li C, Guo M, Xu Y, Sun X (2019). The kinetic changes of systemic inflammatory factors during bevacizumab treatment and its prognostic role in advanced non-small cell lung cancer patients. J Cancer.

[24] Alvarez Secord A, Bell Burdett K, Owzar K, Tritchler D, Sibley AB, Liu Y (2020). Predictive blood-based biomarkers in patients with epithelial ovarian cancer treated with carboplatin and paclitaxel with or without bevacizumab: results from GOG-0218. Clin Cancer Res Off J Am Assoc Cancer Res.

[25] Choe G, Park JK, Jouben-Steele L, Kremen TJ, Liau LM, Vinters HV (2002). Active matrix metalloproteinase 9 expression is associated with primary glioblastoma subtype. Clin Cancer Res Off J Am Assoc Cancer Res.

[26] Liu M-F, Hu Y-Y, Jin T, Xu K, Wang S-H, Du G-Z (2015). Matrix metalloproteinase-9/neutrophil gelatinase-associated lipocalin complex activity in human glioma samples predicts tumor presence and clinical prognosis. Dis Mark.

[27] Hormigo A, Gu B, Karimi S, Riedel E, Panageas KS, Edgar MA (2006). YKL-40 and matrix metalloproteinase-9 as potential serum biomarkers for patients with high-grade gliomas. Clin Cancer Res Off J Am Assoc Cancer Res.

[28] Takano S, Mashiko R, Osuka S, Ishikawa E, Ohneda O, Matsumura A (2010). Detection of failure of bevacizumab treatment for malignant glioma based on urinary matrix metalloproteinase activity. Brain Tumor Pathol.

[29] Lee S, Lee E, Ko E, Ham M, Lee HM, Kim E-S (2018). Tumor-associated macrophages secrete CCL2 and induce the invasive phenotype of human breast epithelial cells through upregulation of ERO1-α and MMP-9. Cancer Lett.

[30] Delire B, Henriet P, Lemoine P, Leclercq IA, Stärkel P (2018). Chronic liver injury promotes hepatocarcinoma cell seeding and growth, associated with infiltration by macrophages. Cancer Sci.

[31] Vannitamby A, Seow HJ, Anderson G, Vlahos R, Thompson M, Steinfort D (2017). Tumour-associated neutrophils and loss of epithelial PTEN can promote corticosteroid-insensitive MMP-9 expression in the chronically inflamed lung microenvironment. Thorax.

[32] Li T-J, Jiang Y-M, Hu Y-F, Huang L, Yu J, Zhao L-Y (2017). Interleukin-17-producing neutrophils link inflammatory stimuli to disease progression by promoting angiogenesis in gastric cancer. Clin Cancer Res Off J Am Assoc Cancer Res.

[33] Wang J, Su X, Yang L, Qiao F, Fang Y, Yu L (2016). The influence of myeloid-derived suppressor cells on angiogenesis and tumor growth after cancer surgery. Int J Cancer.

[34] Hu F, Ku M-C, Markovic D, Dzaye ODA, Lehnardt S, Synowitz M (2014). Glioma-associated microglial MMP9 expression is upregulated by TLR2 signaling and sensitive to minocycline. Int J Cancer.

[35] Gjorgjevski M, Hannen R, Carl B, Li Y, Landmann E, Buchholz M (2019). Molecular profiling of the tumor microenvironment in glioblastoma patients: correlation of microglia/macrophage polarization state with metalloprotease expression profiles and survival. Biosci Rep.

[36] Nozawa H, Chiu C, Hanahan D (2006). Infiltrating neutrophils mediate the initial angiogenic switch in a mouse model of multistage carcinogenesis. Proc Natl Acad Sci USA.

[37] Deryugina EI, Zajac E, Juncker-Jensen A, Kupriyanova TA, Welter L, Quigley JP (2014). Tissue-infiltrating neutrophils constitute the major in vivo source of angiogenesis-inducing MMP-9 in the tumor microenvironment. Neoplasia NYN.

[38] Christoffersson G, Vågesjö E, Vandooren J, Lidén M, Massena S, Reinert RB (2012). VEGF-A recruits a proangiogenic MMP-9-delivering neutrophil subset that induces angiogenesis in transplanted hypoxic tissue. Blood.

[39] Wirsching H-G, Arora S, Zhang H, Szulzewsky F, Cimino PJ, Quéva C (2019). Cooperation of oncolytic virotherapy with VEGF-neutralizing antibody treatment in IDH wildtype glioblastoma depends on MMP9. Neuro-Oncol.

[40] Lee S, Jilani SM, Nikolova GV, Carpizo D, Iruela-Arispe ML (2005). Processing of VEGF-A by matrix metalloproteinases regulates bioavailability and vascular patterning in tumors. J Cell Biol.

