# Supplementary material for: Plasmatic MMP9 released from tumor-infiltrating neutrophils is predictive for bevacizumab efficacy in glioblastoma patients: an AVAglio ancillary study
Source: Acta Neuropathol Commun. 2022 Jan 3;10:1. doi: 10.1186/s40478-021-01305-4 (PMC8722051; doi:10.1186/s40478-021-01305-4)
Supplement: Supplementary file 1 — Additional file 1. Supplemental figures. [file 40478_2021_1305_MOESM1_ESM.pdf]

Supplementary Figure 1: Cohort and sample collection design.

Bev: bevacizumab; TMZ: temozolomide; Plb: placebo

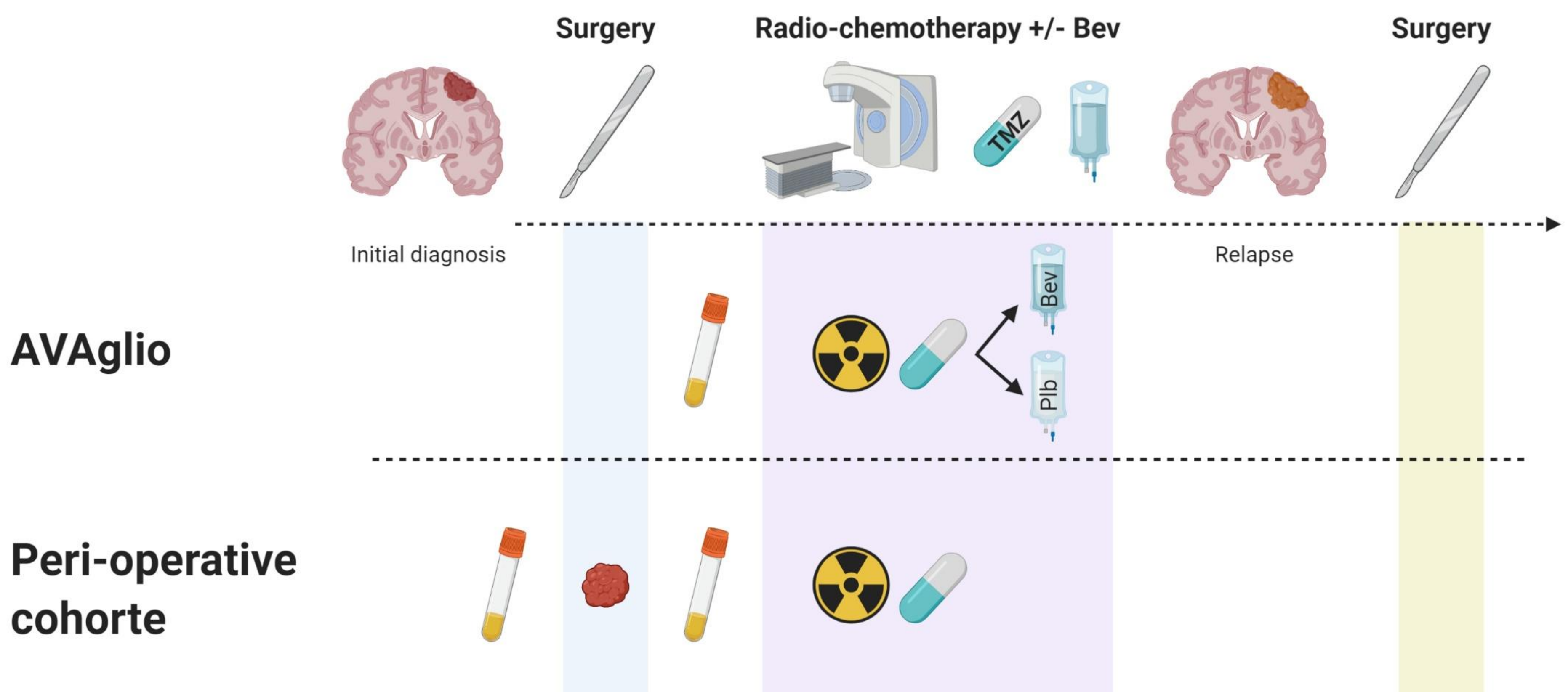

Supplementary figure 2

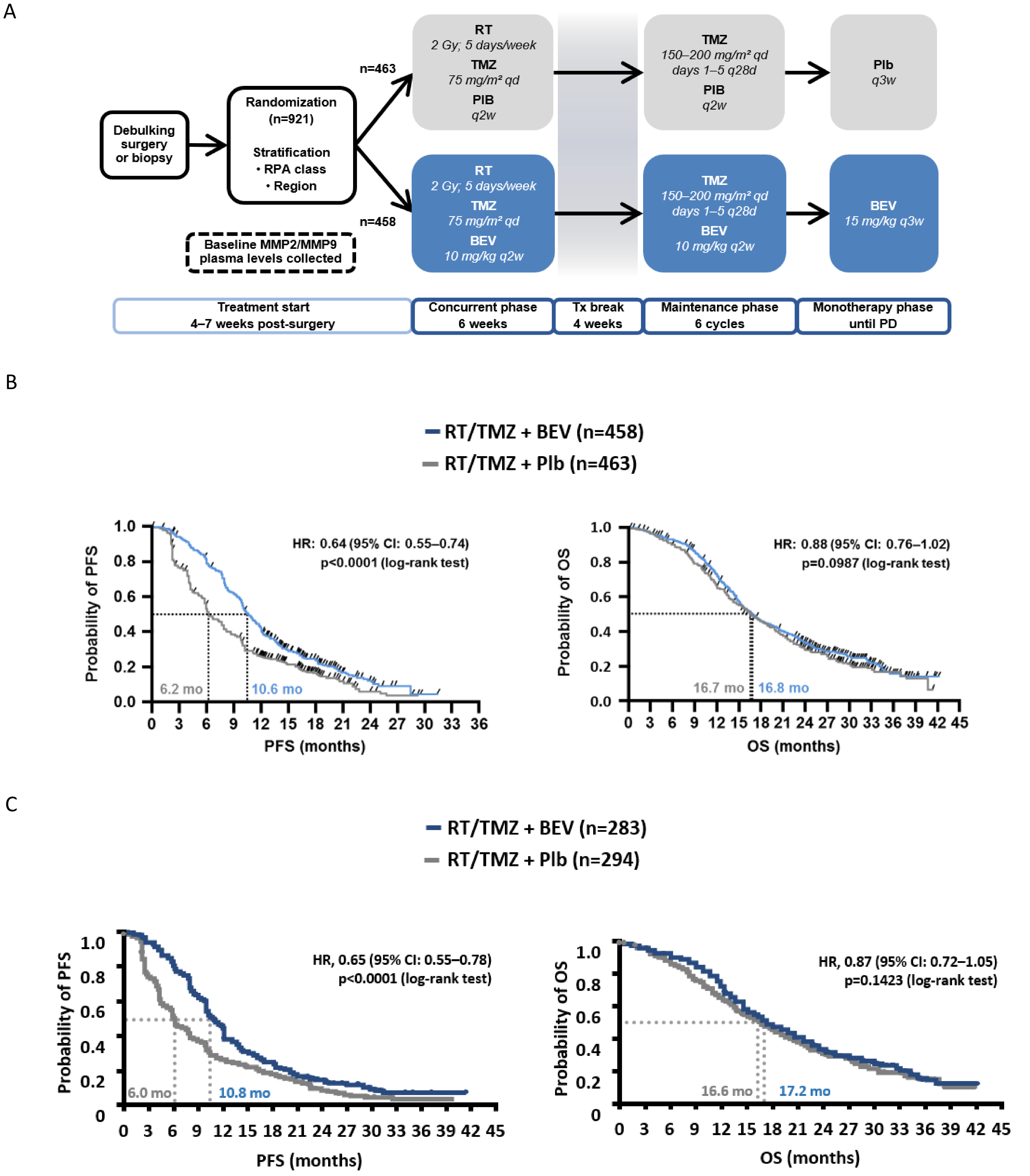

Supplementary Figure 2: AVAGLIO phase III clinical trial. A-AVAGLIO clinical trial design. B-Progression Free Survival and Overall Survival according to treatment arms in the whole trial cohort (921 patients). C- Progression Free Survival and Overall Survival according to treatment arms in the subgroups analyzed in the ancillary study (577 patients). No difference was observed between these two patient groups (original and ancillary). RT: radiotherapy; TMZ: temozolomide; Bev: bevacizumab; Plb: placebo

Supplementary figure 3

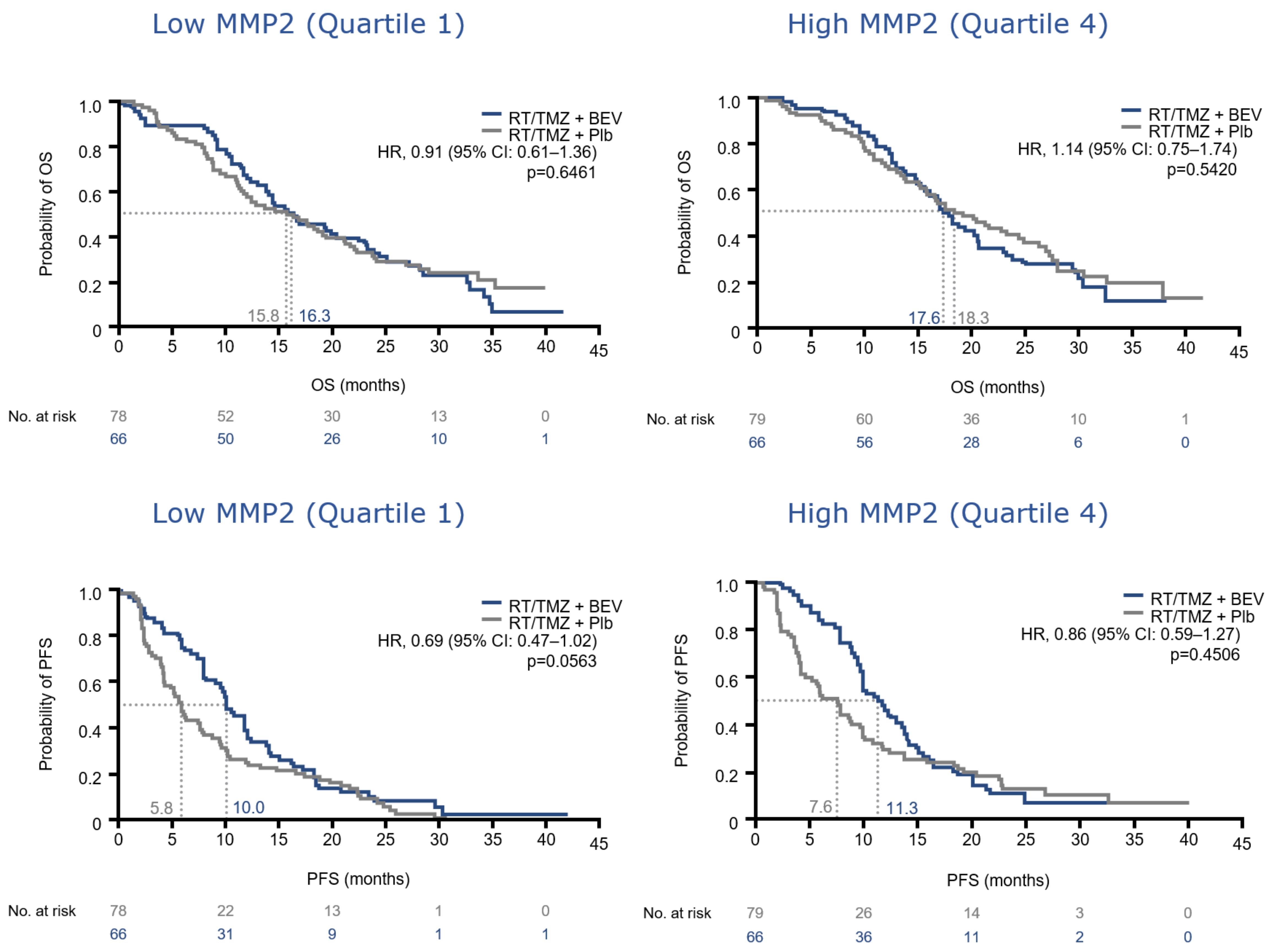

Supplementary Figure 3: Overall Survival (OS, top) and Progression-Free Survival (PFS, bottom) and according to MMP2 plasma level at baseline. RT: radiotherapy; TMZ: temozolomide; Bev: bevacizumab; Plb: placebo

Supplementary Figure 4 –Plasmatic MMP2 and MMP9 activity. Zymogram (A) and quantification (B) of MMP2 and MMP9 activity in plasma before and after surgery in patients with newly-diagnosed glioblastoma, cerebral aneurysm or healthy controls

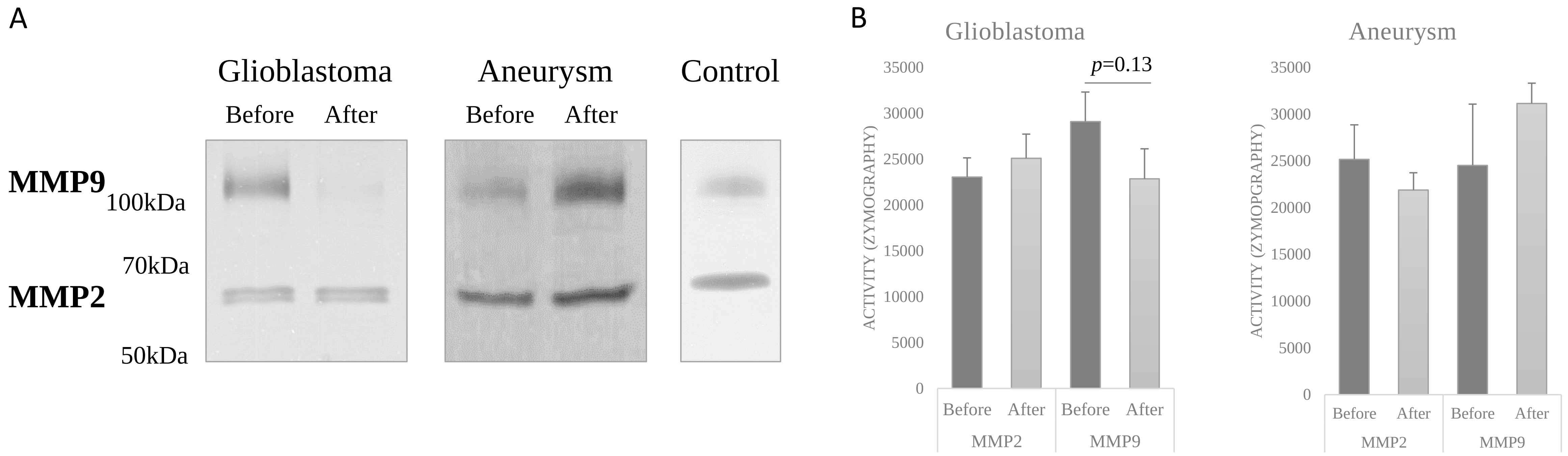

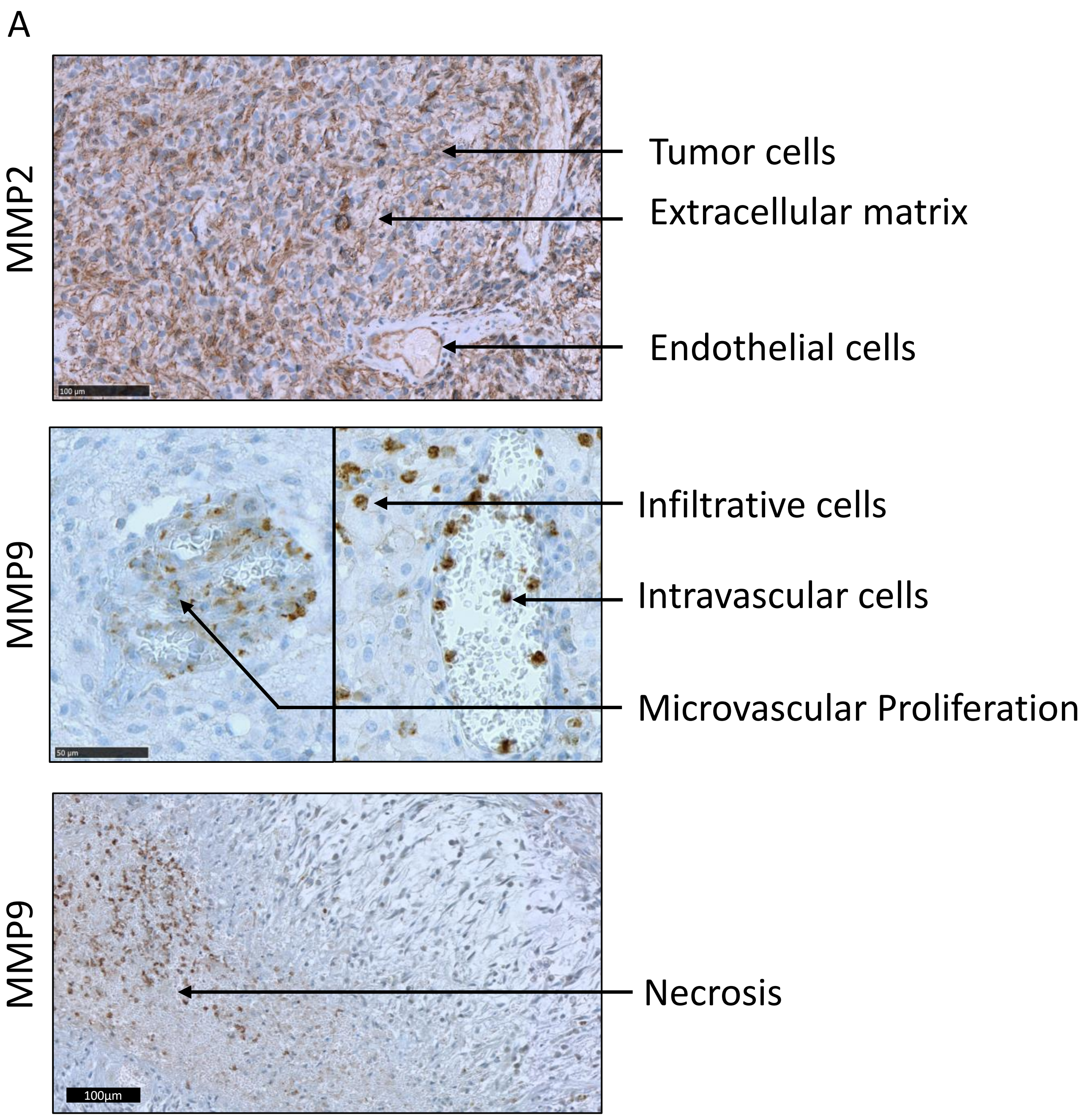

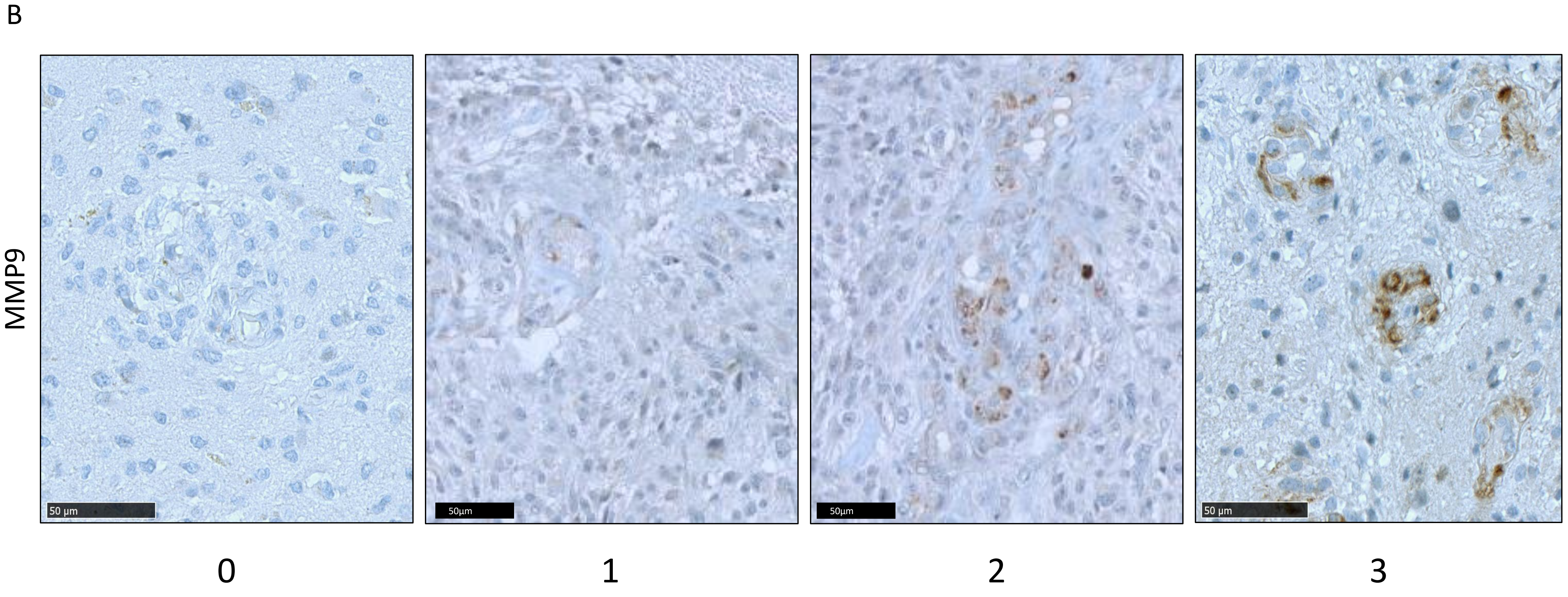

Supplementary figure 5 – Immunostaining illustration. A – Representative examples of regions of interest. B – Representative examples of immunostaining scores on microvascular proliferation.

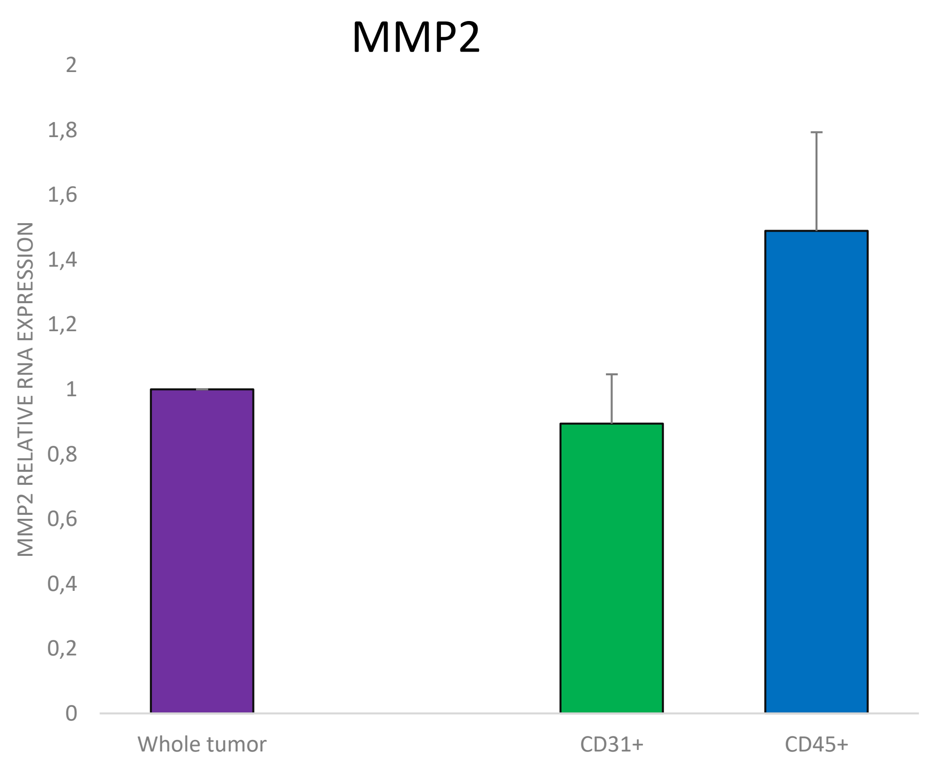

Supplementary Figure 6: MMP2 mRNA expression in CD31+ and CD45+ fraction cells. No difference was observed.

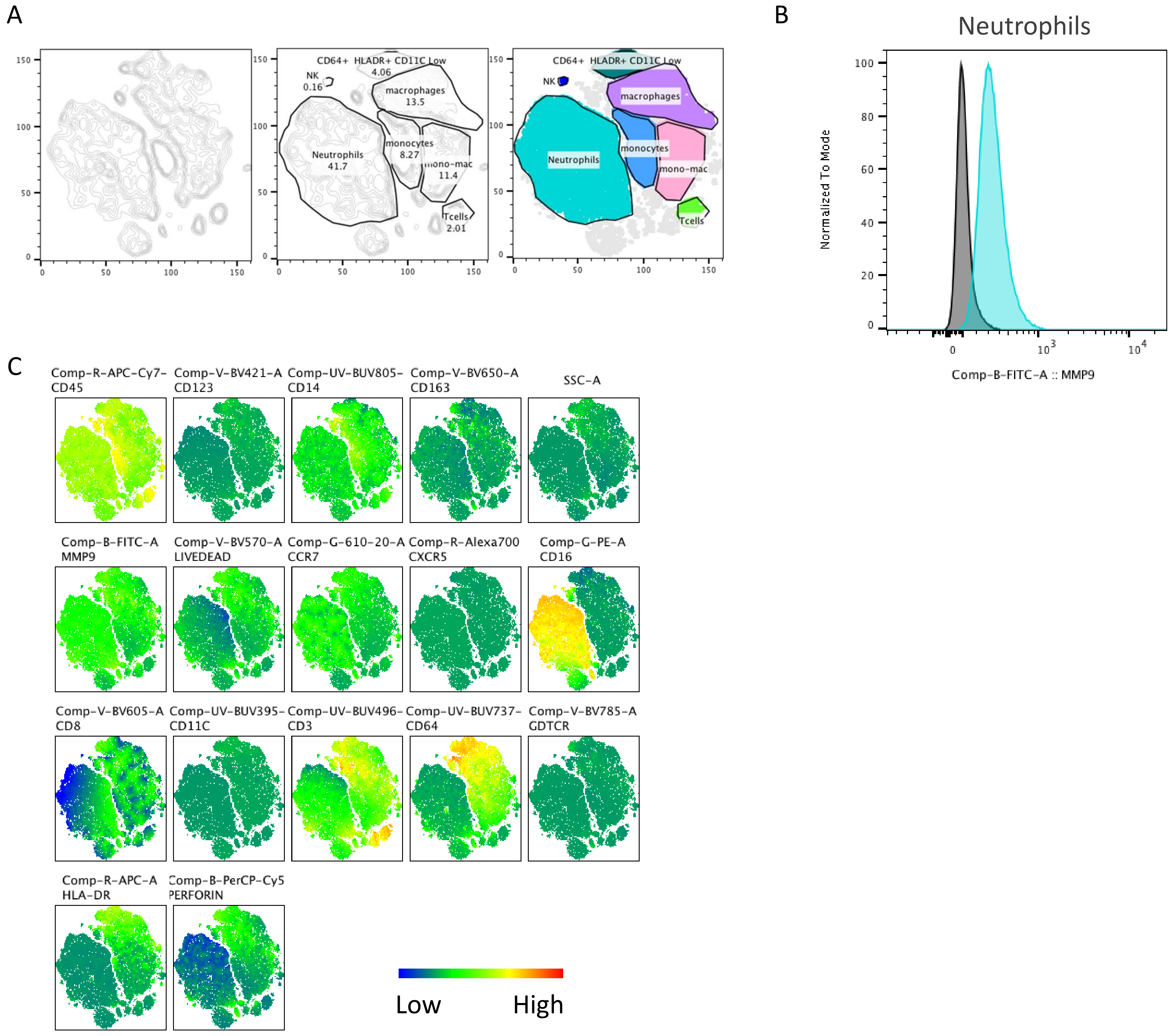

Supplementary Figure 7 – MMP9 is expressed by tumor-infiltrating CD16+ neutrophils: a second *IDHwt* GBM illustration (GBM2). A- T-Sne representation of immune subset. B - MMP9 mean fluorescence intensity (MFI) shift between stained and unstained neutrophils. C - T-Sne representation of each marker including MMP9.
