# Supplementary material for: Plasmatic MMP9 released from tumor-infiltrating neutrophils is predictive for bevacizumab efficacy in glioblastoma patients: an AVAglio ancillary study
Source: Acta Neuropathol Commun. 2022 Jan 3;10:1. doi: 10.1186/s40478-021-01305-4 (PMC8722051; doi:10.1186/s40478-021-01305-4)
Supplement: Supplementary file 2 — Additional file 2. Supplementary tables. [file 40478_2021_1305_MOESM2_ESM.docx]

Supplementary Table 1 – Primers of genes analyzed by RT-qPCR.

| **Gene** | **Forward primer** | **Reverse Primer** |
| --- | --- | --- |
| MMP2 | CCCTGTCACTCCTGAGATCTGC | CACAGTCCGCCAAATGAACC |
| MMP9 | CCTGGAGACCTGAGAACCAA | AGTGTAACCATAGCGGTACAGG |
| 18S | CTACCACATCCAAGGAAGGCA | TTTTTCGTCAACTACCTCCCCG |
| B-actin | CCACACTGTGCCCATCTACG | AGGATCTTCATGAGGTAGTCAGTCAG |
| GAPDH | CAAATTCCATGGCACCGTC | CCCACTTGATTTTGGAGGGA |

Supplementary Table 2 – Patient characteristics of the AVAglio cohort according MMP9 quartiles: bevacizumab arm.

| **Bevacizumab** | | | | | |
| --- | --- | --- | --- | --- | --- |
| **Characteristic** | **Quartile 1**  **(n=72)** | **Quartile 2**  **(n=68)** | **Quartile 3**  **(n=80)** | **Quartile 4**  **(n=63)** | **All**  **(n=283)** |
| **MMP9 plasma level**  Median  Q1–Q3 | 30.0  25.8–39.1 | 64.9  55.2–75.0 | 103.9  92.3–118.0 | 211.8  166.4–296.7 | 83.6  43.8–133.8 |
| **Median age, years (range)** | 58.0 (26–78) | 55.0 (28−76) | 57.0 (28−79) | 59.0 (20−77) | 57.0 (20−79) |
| **Age group, n (%)**  ≥70 years | 7 (10) | 5 (7) | 6 (8) | 9 (14) | 27 (10) |
| **Male, n (%)** | 39 (54) | 46 (68) | 55 (69) | 41 (65) | 181 (64) |
| **WHO performance status, n (%)**  0  1−2 | 39 (54)  33 (46) | 39 (57)  29 (43) | 44 (55)  36 (45) | 29 (46)  34 (54) | 151 (53)  132 (47) |
| **Surgery type, n (%)**  Biopsy only  Partial resection  Complete resection | 2 (3)  38 (53)  32 (44) | 8 (12)  28 (41)  32 (47) | 10 (13)  34 (43)  36 (45) | 13 (21)  31 (49)  19 (30) | 33 (12)  131 (46)  119 (42) |
| **RPA class – CRF, n (%)**  III  IV  V | 13 (18)  45 (63)  14 (19) | 14 (21)  38 (56)  16 (24) | 10 (13)  49 (61)  21 (26) | 9 (14)  35 (56)  19 (30) | 46 (16)  167 (59)  70 (25) |
| **MGMT gene promoter status, n (%)**  Methylated  Non-methylated  Missing | 26 (36)  32 (44)  14 (19) | 17 (25)  39 (57)  12 (18) | 12 (15)  50 (63)  18 (23) | 13 (21)  29 (46)  21 (33) | 68 (24)  150 (53)  65 (23) |
| **Karnofsky performance status at baseline, n (%)**  50−80  90−100 | 19 (26)  53 (74) | 21 (31)  47 (69) | 23 (29)  57 (71) | 21 (33)  42 (67) | 84 (30)  199 (70) |
| **MMSE score, n (%)**  <27  ≥27  Missing | 13 (18)  57 (79)  2 (3) | 12 (18)  56 (82)  0 | 16 (20)  62 (78)  2 (3) | 18 (29)  45 (71)  0 | 59 (21)  220 (78)  4 (1) |
| **Patients taking corticosteroids at baseline, n (%)**  On  Off  Missing | 16 (22)  56 (78)  0 | 19 (28)  49 (72)  0 | 31 (39)  49 (61)  0 | 42 (67)  19 (30)  2 (3) | 108 (38)  173 (61)  2 (1) |

Supplementary Table 3 - Patient characteristics of the AVAglio cohort according MMP9 quartiles: placebo arm.

| **Placebo** | | | | | |
| --- | --- | --- | --- | --- | --- |
| **Characteristic** | **Quartile 1**  **(n=72)** | **Quartile 2**  **(n=76)** | **Quartile 3**  **(n=64)** | **Quartile 4**  **(n=82)** | **All**  **(n=294)** |
| **MMP9 plasma level**  Median  Q1–Q3 | 29.4  21.5–36.5 | 62.9  54.0–69.8 | 111.7  98.4–123.1 | 225.0  174.7–299.5 | 82.4  44.8–160.3 |
| **Median age, years (range)** | 58.0 (24–74) | 58.0 (29–73) | 56.0 (36–78) | 55.5 (21–79) | 56 (21–79) |
| **Age group, n (%)**  ≥70 years | 8 (11) | 4 (5) | 3 (5) | 9 (11) | 24 (8) |
| **Male, n (%)** | 42 (58) | 52 (68) | 36 (56) | 57 (70) | 187 (64) |
| **WHO performance status, n (%)**  0  1−2  Missing | 40 (56)  32 (44)  0 | 41 (54)  35 (46)  0 | 37 (58)  27 (42)  0 | 44 (54)  37 (45)  1 (1) | 162 (55)  131 (45)  1 (<1) |
| **Surgery type, n (%)**  Biopsy only  Partial resection  Complete resection | 1 (1)  44 (61)  27 (38) | 7 (9)  31 (41)  38 (50) | 2 (3)  34 (53)  28 (44) | 11 (13)  49 (60)  22 (27) | 21 (7)  158 (54)  115 (39) |
| **RPA class – CRF, n (%)**  III  IV  V  Missing | 12 (17)  46 (64)  14 (19)  0 | 13 (17)  46 (61)  17 (22)  0 | 6 (9)  43 (67)  15 (23)  0 | 15 (18)  46 (56)  20 (24)  1 (1) | 46 (16)  181 (62)  66 (22)  1 (<1) |
| **MGMT gene promoter status, n (%)**  Methylated  Non-methylated  Missing | 20 (28)  38 (53)  14 (19) | 17 (22)  39 (51)  20 (26) | 14 (22)  38 (59)  12 (19) | 27 (33)  36 (44)  19 (23) | 78 (27)  151 (51)  65 (22) |
| **Karnofsky performance status at baseline, n (%)**  50−80  90−100  Missing | 19 (26)  53 (74)  0 | 26 (34)  50 (66)  0 | 19 (30)  45 (70)  0 | 20 (24)  61 (74)  1 (1) | 84 (29)  209 (71)  1 (<1) |
| **MMSE score, n (%)**  <27  ≥27  Missing | 16 (22)  56 (78)  0 | 15 (20)  61 (80)  0 | 16 (25)  48 (75)  0 | 19 (23)  62 (76)  1 (1) | 66 (22)  227 (77)  1 (<1) |
| **Patients taking corticosteroids at baseline, n (%)**  On  Off  Missing | 16 (22)  56 (78)  0 | 28 (37)  47 (62)  1 (1) | 34 (53)  30 (47)  0 | 57 (70)  24 (29)  1 (1) | 135 (46)  157 (53)  2 (1) |
